# Anesthetic management in cesarean delivery of women with placenta previa: a retrospective cohort study

**DOI:** 10.1186/s12871-021-01472-w

**Published:** 2021-10-19

**Authors:** Dazhi Fan, Jiaming Rao, Dongxin Lin, Huishan Zhang, Zixing Zhou, Gengdong Chen, Pengsheng Li, Wen Wang, Ting Chen, Fengying Chen, Yuping Ye, Xiaoling Guo, Zhengping Liu

**Affiliations:** 1 Foshan Fetal Medicine Research Institute, Affiliated Foshan Women and Children Hospital, Southern Medical University, Foshan, 528000 Guangdong China; 2 Department of Obstetrics, Affiliated Foshan Women and Children Hospital, Southern Medical University, Foshan, 528000 Guangdong China; 3 Department of Foetal Ultrasonic, Affiliated Foshan Women and Children Hospital, Southern Medical University, Foshan, 528000 Guangdong China; 4 Department of Radiology, Affiliated Foshan Women and Children Hospital, Southern Medical University, Foshan, 528000 Guangdong China; 5 Department of Anesthesiology, Affiliated Foshan Women and Children Hospital, Southern Medical University, Foshan, 528000 Guangdong China

**Keywords:** Placenta previa, General anesthesia, Neuraxial anesthesia, Cohort

## Abstract

**Background:**

The incidence of placenta preiva is rising. Cesarean delivery is identified as the only safe and appropriate mode of delivery for pregnancies with placenta previa. Anesthesia is important during the cesarean delivery. The aim of this study is to assess maternal and neonatal outcomes of patients with placenta previa managed with neuraxial anesthesia as compared to those who underwent general anesthesia during cesarean delivery.

**Methods:**

A retrospective cohort study was performed of all patients with placenta preiva at our large academic institution from January 1, 2014 to June 30, 2019. Patients were managed neuraxial anesthesia and general anesthesia during cesarean delivery.

**Results:**

We identified 1234 patients with placenta previa who underwent cesarean delivery at our institution. Neuraxial anesthesia was performed in 737 (59.7%), and general anesthesia was completed in 497 (40.3%) patients. The mean estimated blood loss at neuraxial anesthesia of 558.96 ± 42.77 ml were significantly lower than the estimated blood loss at general anesthesia of 1952.51 ± 180 ml (*p* < 0.001). One hundred and forty-six of 737 (19.8%) patients required blood transfusion at neuraxial anesthesia, whereas 381 out of 497 (76.7%) patients required blood transfusion at general anesthesia. The rate neonatal asphyxia and admission to NICU at neuraxial anesthesia was significantly lower than general anesthesia (2.7% vs. 19.5 and 18.2% vs. 44.1%, respectively). After adjusting confounding factors, blood loss was less, Apgar score at 1- and 5-min were higher, and the rate of blood transfusion, neonatal asphyxia, and admission to NICU were lower in the neuraxial group.

**Conclusions:**

Our data demonstrated that neuraxial anesthesia is associated with better maternal and neonatal outcomes during cesarean delivery in women with placenta previa.

**Supplementary Information:**

The online version contains supplementary material available at 10.1186/s12871-021-01472-w.

## Background

Placenta previa is characterized by the abnormal implantation of placental tissue overlying the endocervical os [1]. It is associated with severe maternal and fetal morbidity and mortality [2]. The strongest risk factor for placenta previa is previous cesarean deliveries [3]. Along with the increasing rate of cesarean delivery, the incidence of placenta previa is increasing, and it is estimated be 1 in 200 pregnancies worldwide [4] and 1.24% in Chinese pregnancy women [5].

Cesarean delivery is identified as the only safe and appropriate mode of delivery for pregnancies with placenta previa [1]. The key role of anesthetist is provision of a safe, comfortable and positive birth experience for pregnant women and an optimal operating condition for obstetricians during cesarean delivery [6]. General anesthesia is a more quickly administered procedure and is preferred in cases where speed is important [7]. However, some early studies identified an association between general anesthesia for cesarean delivery and increased rates of airway complications, including failed intubations, maternal aspiration and aspiration pneumonitis [8, 9]. Neuraxial anesthesia can cause a substantial drop in maternal blood pressure, which may affect both mother and fetus, and may be dangerous when the woman has a bleeding complication [10, 11]. The advantages of neuraxial anesthesia include reduction in uteroplacental drug transfer, avoidance airway instrumentation, and improvement parent-baby bonding via immediate skin to skin contact, since the mother is awake during the procedure [12].

Because of the possible increased blood loss in these patients, some believe that general anesthesia is preferable for cesarean delivery for placenta previa, while others believe that cesarean delivery for placenta previa can be usually safely performed using neuraxial anesthesia [1, 13]. The RCOG considered that neuraxial anesthesia is safe and had a lower risk of hemorrhage than general anesthesia for cesarean delivery in women with placenta previa [3]. Due to the relatively uncommon occurrence of placenta previa, larger cohort data regarding characteristics and outcomes of placenta previa cases undergoing cesarean delivery with anesthesia mode are limited. This study aimed to describe the association between anesthetic technique (neuraxial vs. general) and blood loss and maternal intraoperative hemodynamics in patients undergoing cesarean delivery for placenta previa in a large cohort database. These findings may be beneficial for the anesthesia risk stratification, counseling, and delivery planning of women diagnosed with placenta previa.

## Methods

We performed a retrospective cohort study between the years 2014–2019 in a high-volume delivery suite in China, which is a tertiary referral medical center with approximately 13,000 deliveries each year [14]. The study was approved by the institutional review board (number FSFY-MEC-2019-044) and was conducted in accordance with the ethical standards described in an appropriate version of the 1975 Declaration of Helsinki, as revised in 2000.

Pregnant women who met the following inclusion criteria were included for analysis: 1) placenta preiva diagnosed by ultrasound before delivery; 2) placenta previa confirmed during delivery by obstetrician; 3) pregnant women undergoing cesarean delivery; 4) singleton gestation. Placenta previa was diagnosed using the last trans-vaginal or -abdominal ultrasonography performed before delivery; transvaginal ultrasonography was preferred if the placenta was located in the posterior wall of the uterus. Trained physicians recorded the distance from the leading placental edge to the internal cervix os, and the placenta covers the cervical os [3]. Women whose pregnancies were terminated or who delivered before 27w6d were excluded from the cohort. Marginal placenta previa pregnancy women were also excluded. Patients with placenta accreta spectrum (PAS) were confirmed during surgery by clinical assessment of the surgical team and by histopathological examination after cesarean hysterectomy or uterus tissue.

Cesarean delivery was performed under general or neuraxial anesthesia. The choice of anesthesia method is determined by consultation between the obstetrician and the anesthesiologist, according to the patient’s background and contraindications. The anesthetic was chosen by the anesthetist’s preference. General anesthesia was performed with propofol (1.0 mg/kg), rocuronium bromide (0.6 mg/kg), and 3% sevofulrane (30 ml) followed by tracheal intubation and mechanical ventilation just before skin incision. Preparations for rapid blood and fluid replacement were made in all patients before surgery. Continuous pumping of propofol (1.0 mg/kg) and sufentanil (0.3 μg/kg) was performed to maintain the depth of anesthesia. Patients were preoxygenated with 100% oxygen via face mask for 2 min before induction. For neuraxial anesthesia, a 25-gauge pencil-point spinal needle is used to access the spinal space at the level of L2–3 or L3–4. Upon return of cerebrospinal fluid, 0.5% bupivacaine (12 mg) was injected. According to the needs of the operation, 2.0% ropivacaine was added to maintain intraoperative anesthesia.

All patients received oxytocin 20 units and carbetocin 100 μg intravenously drip immediately after delivery of the placenta to reducing the postpartum hemorrhage. Misoprostol 500 mg rectal and/or hemabate 250 mg intramuscular injection were given when the obstetrician complained of a noticeable bleeding in the lower part of the uterus following removal of the placenta.

Patients were identified from a prospective database of all patients with a diagnosis of placenta previa made during the study period. The data were collected retrospectively from the medical record after discharge. The database was updated every two weeks, and there were special personnel for maintenance and sampling inspection. Data was acquired using relevant electronic health record data including demographics, pregnancy characteristics, pathology findings, anesthesia method (general or neuraxial), operative time, anesthesia-to-delivery time, blood loss, hemoglobin concentration, Apgar score (1 min, 5 min, and 10 min), neonatal asphyxia, and admission to NICU (neonatal intensive care unit). The primary outcome was estimated blood loss (EBL). Blood loss was collected and measured using a drape with a blood collection system around the abdominal wound from the abdominal cavity during the cesarean delivery. Gauzes were used to collect blood from the vagina. All gauzes with blood were collected, weighed and an equivalent volume was calculated. The volume of blood loss is equal to the weight of blood loss ÷ 1.05. Any post-cesarean delivery blood loss was also quantified [15]. Secondary outcomes were transfusion blood rate, Apgar, and NICU. Blood transfusion during cesarean delivery was performed by the clinician in accordance with protocol.

Statistical analysis was completed using SPSS 21.0. Statistical assessment of our data was performed using descriptive statistics as well as *t*-tests, Wilcoxon rank-sum and chi-square test for continuous and categorical variables, respectively. Univariate analysis was performed to determine the role of the type of anesthesia in the outcomes, unadjusted odds ratios or beta coefficients, 95% confidence intervals, and 2-side *p* values were calculated. Multivariate logistic or line regressions were further performed, and adjusted odds ratios or beta coefficients were calculated, as well. Variables with a *p*-value < 0.05 in the univariate analysis were entered into the multivariate model. Potential confounders included gestational weeks, gravity, PAS, anterior placenta, previous cesarean delivery, previous placenta previa, antepartum hemorrhage, emergency cesarean delivery, and anesthesia-to-delivery time (min). Given that management for PAS cases is different from that for placenta previa, the results were re-calculated after excluding those cases with placenta previa complication with PAS.

## Results

A total of 1234 placenta previa subjects were included in the study; 737 (59.7%) with neuraxial anesthesia and 497 (40.3%) with general anesthesia. Table 1 summarized the baseline distribution of placenta previa subjects. The neuraxial and general groups were similar in maternal age, height, weight, and BMI. Subjects with general anesthesia were delivered earlier, had more gravidities, and had a higher proportion of placenta accreta spectrum, anterior placenta, antepartum hemorrhage, emergency cesarean delivery, and history of cesarean delivery and placenta previa.

**Table 1:** Maternal characteristics of among included patients

	Total	Neuraxial group (*n* = 737)	General group (*n* = 497)	t/z/*χ*^*2*^	p
Age (year)	32.62 ± 5.14	32.47 ± 5.20	32.83 ± 5.04	1.182	0.237
Height (cm)	157.45 ± 4.90	157.60 ± 4.89	157.23 ± 4.91	1.294	0.196
Weight (kg)	64.99 ± 8.86	65.04 ± 8.75	64.91 ± 9.03	0.244	0.808
BMI (kg/m^2^)	26.21 ± 3.40	26.17 ± 3.40	26.27 ± 3.41	0.506	0.613
Gestational age (wk)	36.44 ± 2.41	36.83 ± 2.47	35.87 ± 2.20	7.120	0.001
Preterm labor (< 37 weeks)	658 (53.3%)	313 (42.5%)	345 (69.4%)	86.599	0.001
Gravity	3 (2–4)	2 (2–3)	3 (2–4)	6.822	0.001
Placenta accreta spectrum	252 (20.4%)	73 (9.9%)	179 (36.0%)	124.531	0.001
Anterior placenta	521 (42.2%)	228 (30.9%)	293 (59.0%)	95.515	0.001
Previous cesarean delivery	552 (44.7%)	224 (30.4%)	328 (66.0%)	152.187	0.001
Previous placenta previa	116 (9.4%)	53 (7.2%)	63 (12.7%)	10.485	0.001
Antepartum hemorrhage	493 (40.0%)	268 (36.4%)	225 (45.3%)	9.818	0.002
Emergency cesarean delivery	365 (29.6%)	237 (32.2%)	128 (25.8%)	5.842	0.016

Values are mean ± SD, median (interquartile range) or number of subjects

*BMI* Body mass index (kg/m^2^)

Table 2 showed the perioperative data and maternal and neonatal outcomes between the two groups. Estimated blood loss was less (558.96 ± 42.77 ml vs. 1952.51 ± 180.00 ml) and the rate of blood transfusion was lower in the neuraxial group. The preoperative hemoglobin concentration was higher in the general group. However, the postoperative hemoglobin concentration was not different between the two groups. The operating time and anesthesia-to-delivery time were shorter in the neuraxial group. For neonatal outcomes, the Apgar scores were all higher at 1-, 5-, and 10-min in the neuraxial group, and the proportion of neonatal asphyxia and admission to NICU were lower in the neuraxial group.

**Table 2:** Perioperative data and maternal and neonatal outcomes

	Total	Neuraxial group (n = 737)	General group (n = 497)	t/*χ*^*2*^	p
Estimated blood loss (mL)	1121.90 ± 137.27	558.96 ± 42.77	1952.51 ± 180.00	16.819	0.001
Blood Transfusion	527 (42.7%)	146 (19.8%)	381 (76.7%)	392.075	0.001
Hemoglobin concentration (g/L)					
Preoperative values	105.84 ± 15.90	107.93 ± 15.02	102.65 ± 16.69	5.624	0.001
Postoperative values	101.60 ± 29.48	100.71 ± 15.41	102.89 ± 42.20	0.136	0.214
Operating time (min)	73.39 ± 5.58	53.83 ± 2.75	102.87 ± 7.22	14.314	0.001
Anesthesia-to-delivery time (min)	34.30 ± 2.86	29.92 ± 2.16	40.86 ± 3.57	6.680	0.001
Hysterectomy	18 (1.5%)	2 (0.3%)	16 (3.2%)	17.946	0.001
Apgar score (1 min)	10 (10–10)	10.0 (10–10)	8 (7–10)	21.685	0.001
Apgar score (5 min)	10 (10–10)	10.0 (10–10)	10 (9.0–10)	11.462	0.001
Apgar score (10 min)	10 (10.0–10)	10 (10–10)	10 (10–10)	6.926	0.001
Asphyxia_neonatal	117 (9.5%)	20 (2.7%)	97 (19.5%)	97.655	0.001
Admission to NICU	353 (28.6%)	134 (18.2%)	219 (44.1%)	97.365	0.001

*NICU* neonatal intensive care unit

In the regression models, blood loss was less, and preoperative hemoglobin concentration and Apgar score were higher, and the rate of blood transfusion, neonatal asphyxia, and admission to NICU were lower in the neuraxial group. After adjusting anesthesia-to-delivery time, there was no substantial change in the results. After further adjusting for anesthesia-to-delivery time and other relevant confounding factors (gestational weeks, gravity, PAS, anterior placenta, previous cesarean delivery, previous placenta previa, antepartum hemorrhage, and emergency cesarean delivery), we found that the above results remained significantly (Table 3). After excluding PAS cases, the main results did not materially change, either (Supplement Tables 1, 2 and 3).

**Table 3:** Regression analysis for factors affecting maternal and neonatal outcomes (neuraxial vs. general)

	OR/*β* (95%CI)	P	OR/*β* (95%CI)^a^	P	OR/*β* (95%CI)^b^	P
Estimated blood loss (mL)	− 1393.55 (− 1530.22 to − 1256.87)	0.001	− 1277.10 (− 1412.71 to − 1141.49)	0.001	− 734.79 (− 901.78 to − 567.79)	0.001
Blood Transfusion	0.08 (0.06 to 0.10)	0.001	0.08 (0.06 to 0.11)	0.001	0.13 (0.09 to 0.18)	0.001
Hemoglobin concentration						
Preoperative values	5.27 (3.43 to 7.11)	0.001	5.54 (3.67 to 7.42)	0.001	2.68 (0.16 to 5.21)	0.037
Postoperative values	−2.18 (−5.63 to 1.26)	0.214	−1.15 (−4.67 to 2.36)	0.520	−0.28 (− 5.47 to 4.92)	0.917
Apgar score (1 min)	1.73 (1.57 to 1.89)	0.001	1.66 (1.50 to 1.82)	0.001	1.29 (1.09 to 1.49)	0.001
Apgar score (5 min)	0.43 (0.33 to 0.54)	0.001	0.39 (0.28 to 0.49)	0.001	0.30 (0.16 to 0.44)	0.001
Apgar score (10 min)	0.15 (0.07 to 0.24)	0.001	0.14 (0.06 to 0.23)	0.001	0.12 (0.01 to 0.23)	0.030
Asphyxia_neonatal	0.12 (0.07 to 0.19)	0.001	0.12 (0.07 to 0.19)	0.001	0.17 (0.09 to 0.32)	0.001
Admission to NICU	0.28 (0.22 to 0.37)	0.001	0.28 (0.21 to 0.36)	0.001	0.39 (0.26 to 0.60)	0.001

^a^Adjusted for anesthesia-to-delivery time (min)

^b^Adjusted for anesthesia-to-delivery time (min), and relevant confounding factors (gestational weeks, gravity, PAS, anterior placenta, previous cesarean delivery, previous placenta previa, antepartum hemorrhage, and emergency cesarean delivery)

## Discussion

In this retrospective analysis of 1234 women with placenta previa, we found that neuraxial anesthesia is associated with several benefits during cesarean delivery in our population, including decreased blood loss, lower need for blood product transfusion, and increased neonatal Apgar score, lower neonatal asphyxia and admission to NICU. We also found anesthesia-to-delivery interval had little influence on the results of the study.

The main strength of the present study is related to the relevant lager sample size in a single center during a relatively short time. Meanwhile, confounding factors were controlled by multivariable analysis to make the results more believable. Further, cases with placenta previa complication with PAS were excluded to recalculate to show the stability of the results. An obvious limitation of the study is its single center retrospective nature and the inherent limitations of retrospective data collection. While we made all efforts to objectively compare anesthesia outcomes between the two groups, it must be acknowledged that the groups likely differed in a priori anesthesia risks. Low-risk patients will be given regional and higher-risk patients a general anesthetic and it is impossible to retrospectively correct for this inevitable bias. In addition, we learned a lot about surgical and anesthetic techniques, as well caring for these patients over the study period and this may have influenced outcomes, including blood loss.

Placenta previa carried a significant risk of antepartum hemorrhage. Our 2017 systematic review and meta-analysis of 29 observational studies found that above half of placenta previa women had antepartum hemorrhage [16]. This cohort finding regarding antepartum hemorrhage is congruent with the previous meta-analysis. Placenta previa women undergoing a general anesthetic have lower preoperative hemoglobin concentration which could be related to their higher incidence of antepartum hemorrhage. Fortunately, there was a little difference in hemoglobin pre- and post-operative and had not found a difference between the postoperative hemoglobin concentration and the two groups. A possible explanation for these findings was that blood transfusion play a big role during labor and delivery. Therefore, adequate blood supply was essential for pregnant women with heavy bleeding and high risk of bleeding, such as placenta previa.

The relationship between anesthesia-to-delivery interval and adverse maternal and neonatal outcomes has been reported in retrospective studies [10, 17]. Delivery within 27 min of anesthesia start was associated with umbilical arterial pH > 7.1, and delivery within 30 min was associated with umbilical arterial pH > 7.0 [17]. In a retrospective cohort study, the authors found that prolonged anesthesia-to-delivery interval was associated with an increased relative risk for neonatal acidosis in planned cesarean deliveries [17].

We found the anesthesia-to-delivery interval was longer in the general anesthesia group. This result was inconsistent with perception. Cystoscopy and separate the adherent abdominal tissue would consume a lot of time in severe patients, such as complication with PAS. That’s why when we excluded patients with PAS, the difference was disappear between the two groups. The general anesthesia women have higher incidence of unfavorable maternal and neonatal outcomes which could be related to their longer anesthesia-to-delivery time. However, after adjusting the anesthesia-to-delivery interval, there has been no real change in the unfavorable maternal and neonatal outcomes between the two groups.

Our data showed that neuraxial anesthesia was associated with better maternal and neonatal outcomes, including less blood loss and transfusion and lower rate of neonatal asphyxia and admission to NICU. Both the patients’ background and the type of anesthesia may have influenced the results. A significantly higher risk of most complications was found in women who had a general anesthesia. The proportion placenta accreta spectrum, anterior placenta and other risk factors are higher in general group. These factors can aggravate maternal and neonatal outcomes [18–21]. In addition, retrospective and prospective studies also suggested that neuraxial anesthesia is associated with less blood loss and transfusion requirements [13, 22, 23]. Hong JY et al. [13] reported that neuraxial anesthesia received a significantly smaller transfusion than the general anesthesia for patients with placenta previa. Frederiksen MC et al. [22] also found neuraxial anesthesia decreased intraoperative blood loss and the need for blood transfusion in women with placenta previa. Meanwhile, Parekh N et al. [23] found neuraxial anesthesia was associated with a significantly reduced estimated blood loss and reduced need for blood transfusion from a larger consecutive placenta previa cases study.

A major limitation of previous studies is lack of control for confounding factors that are also associated with important outcomes such as blood loss. Given the major baseline differences between the two anesthetic groups, we offered the opportunity to assess these factors in detail through multivariable analysis in this a large single-center study. PAS is a very different from placenta previa regarding management. We further excluded placenta previa complication with PAS to evaluate the results and the results did not materially change. These suggested that neuraxial anesthesia was associated with several benefits during cesarean delivery for placenta previa women.

Placenta previa is the most common cause of massive obstetric hemorrhage and is associated with an increased incidence of massive transfusion, prolonged surgery and length of hospital stay [24]. A multi-disciplinary team (including obstetricians, neonatologists, midwives, anesthetists, critical care staff, ect.) should be approached to management of these patients. Placenta previa will become more frequently encountered by obstetric anesthetics in the future.

Both general and neuraxial anesthesia options have advantages and disadvantages for patients with placenta previa. The ideal anesthetic choice for patients with placenta previa should require individualized planning based on patients’, anesthetic and surgical factors. Patient factors include pregnant women’s preference, predicted difficult airway and contraindications to neuraxial anesthesia, and surgical factors include imaging interpretation predicting extensive or prolonged surgery.

## Conclusions

This study presents a paradigm for the anesthetic management of placenta previa that is consistent with current RCOG guidelines and with data presented by other authors. Our study adds to the limited existing literature supporting neuraxial anesthesia is safe and lower risk of hemorrhage for cesarean delivery in women with placenta previa.

## Supplementary Information

**Additional file 1: Table 1**. Maternal characteristics of among included patients (excluding placenta accreta spectrum).

**Additional file 2: Table 2**. Perioperative data and maternal and neonatal outcomes (excluding placenta accreta spectrum).

**Additional file 3: Table 3**. Regression analysis for factors affecting maternal and neonatal outcomes (neuraxial vs. general) (excluding placenta accreta spectrum).

## Data Availability

The datasets used and/or analysed during the current study are available from the corresponding author on reasonable request.

## Abbreviations

CI: Confidence interval
EBL: Estimated blood loss
NICU: Neonatal intensive care unit
OR: Odds ratio
PAS: Placenta accreta spectrum
