# Supplementary material for: Anesthetic management in cesarean delivery of women with placenta previa: a retrospective cohort study
Source: BMC Anesthesiol. 2021 Oct 19;21:247. doi: 10.1186/s12871-021-01472-w (PMC8524954; doi:10.1186/s12871-021-01472-w)
Supplement: Supplementary file 1 — Additional file 1: Table 1. Maternal characteristics of among included patients (excluding placenta accreta spectrum). [file 12871_2021_1472_MOESM1_ESM.docx]

Table 1. Maternal characteristics of among included patients (excluding placenta accreta spectrum).

|  | Total  (n=982) | Neuraxial group (n=664) | General group (n=318) | t/z/*χ^2^* | p |
| --- | --- | --- | --- | --- | --- |
| Age (year) | 32.55 ± 5.12 | 32.51 ± 5.14 | 32.62 ± 5.06 | 0.299 | 0.765 |
| Height (cm) | 157.47 ± 4.90 | 157.63 ± 4.90 | 157.14 ± 4.90 | 1.451 | 0.147 |
| Weight (kg) | 65.03 ± 8.89 | 64.93 ± 8.78 | 65.24 ± 9.12 | 0.483 | 0.629 |
| BMI (kg/m^2^) | 26.22 ± 3.42 | 26.12 ± 3.41 | 26.42 ± 3.45 | 1.259 | 0.209 |
| Gestational age (wk) | 36.51 ± 2.49 | 36.80 ± 2.52 | 35.91 ± 2.30 | 5.265 | 0.001 |
| Preterm labor (<37 weeks) | 491 (50.0%) | 286 (43.1%) | 205 (64.5%) | 39.363 | 0.001 |
| Gravity | 2 (2-3) | 2 (2-3) | 3 (2-4) | 4.832 | 0.001 |
| Anterior placenta | 352 (35.8%) | 197 (29.7%) | 155 (48.7%) | 34.016 | 0.001 |
| Previous cesarean delivery | 376 (38.3%) | 194 (29.2%) | 182 (57.2%) | 71.426 | 0.001 |
| Previous placenta previa | 93 (9.5%) | 50 (7.5%) | 43 (13.5%) | 9.004 | 0.004 |
| Antepartum hemorrhage | 400 (40.7%) | 247 (37.2%) | 153 (48.1%) | 10.610 | 0.001 |
| Emergency cesarean delivery | 318 (32.4%) | 221 (33.3%) | 97 (30.5%) | 0.759 | 0.423 |

Values are mean ± SD, median (interquartile range) or number of subjects.

BMI, Body mass index (kg/m^2^)
