# Supplementary material for: Anesthetic management in cesarean delivery of women with placenta previa: a retrospective cohort study
Source: BMC Anesthesiol. 2021 Oct 19;21:247. doi: 10.1186/s12871-021-01472-w (PMC8524954; doi:10.1186/s12871-021-01472-w)
Supplement: Supplementary file 2 — Additional file 2: Table 2. Perioperative data and maternal and neonatal outcomes (excluding placenta accreta spectrum). [file 12871_2021_1472_MOESM2_ESM.docx]

Table 2. Perioperative data and maternal and neonatal outcomes (excluding placenta accreta spectrum).

|  | Total  (n=982) | Neuraxial group (n=664) | General group (n=318) | t/*χ^2^* | p |
| --- | --- | --- | --- | --- | --- |
| Estimated blood loss (mL) | 766.55 ± 80.59 | 528.73 ± 36.67 | 1261.95 ± 115.99 | 10.925 | 0.001 |
| Blood Transfusion | 331 (33.7%) | 122 (18.4%) | 209 (65.7%) | 215.741 | 0.001 |
| Hysterectomy | 6 (0.6%) | 2 (0.3%) | 4 (1.3%) | 3.241 | 0.090 |
| Hemoglobin concentration (g/L) |  |  |  |  |  |
| Preoperative values | 106.28 ± 16.32 | 107.83 ± 15.23 | 102.96 ± 18.02 | 4.298 | 0.001 |
| Postoperative values | 101.10 ± 16.12 | 100.86 ± 15.54 | 101.59 ± 17.24 | 0.656 | 0.512 |
| Operating time (min) | 60.72 ± 3.78 | 52.56 ± 2.73 | 77.94 ± 4.94 | 8.502 | 0.001 |
| Anesthesia-to-delivery time (min) | 31.07 ± 2.71 | 29.74 ± 2.23 | 33.86 ± 3.51 | 1.901 | 0.058 |
| Apgar score (1 min) | 10 (9-10) | 10 (10-10) | 8 (8-10) | 18.428 | 0.001 |
| Apgar score (5 min) | 10 (10-10) | 10 (10-10) | 10 (10-10) | 9.350 | 0.001 |
| Apgar score (10 min) | 10 (10-10) | 10 (10-10) | 10 (10-10) | 5.837 | 0.001 |
| Asphyxia_neonatal | 70 (7.1%) | 20 (3.0%) | 50 (15.7%) | 52.479 | 0.001 |
| Admission to NICU | 257 (26.2%) | 125 (18.8%) | 132 (41.5%) | 57.264 | 0.001 |

Values are mean ± SD, median (interquartile range) or number of subjects.

NICU: neonatal intensive care unit;
