# Supplementary material for: Anesthetic management in cesarean delivery of women with placenta previa: a retrospective cohort study
Source: BMC Anesthesiol. 2021 Oct 19;21:247. doi: 10.1186/s12871-021-01472-w (PMC8524954; doi:10.1186/s12871-021-01472-w)
Supplement: Supplementary file 3 — Additional file 3: Table 3. Regression analysis for factors affecting maternal and neonatal outcomes (neuraxial vs. general) (excluding placenta accreta spectrum). [file 12871_2021_1472_MOESM3_ESM.docx]

Table 3. Regression analysis for factors affecting maternal and neonatal outcomes (neuraxial vs. general) (excluding placenta accreta spectrum).

|  | OR/*β* (95%CI) | P |  | OR/*β* (95%CI)* | P |  | OR/*β* (95%CI)** | P |
| --- | --- | --- | --- | --- | --- | --- | --- | --- |
| Estimated blood loss (mL) | -733.23 (-831.17 to -635.28) | 0.001 |  | -706.73 (-802.86 to -610.60) | 0.001 |  | -583.61 (-704.19 to -463.02) | 0.001 |
| Blood Transfusion | 0.12 (0.09 to 0.16) | 0.001 |  | 0.12 (0.09 to 0.16) | 0.001 |  | 0.15 (0.10 to 0.22) | 0.001 |
| Hemoglobin concentration |  |  |  |  |  |  |  |  |
| Preoperative values | 4.87 (2.65 to 7.10) | 0.001 |  | 5.02 (2.77 to 7.26) | 0.001 |  | 2.31 (-0.53 to 5.16) | 0.111 |
| Postoperative values | -0.74 (-2.93 to 1.47) | 0.512 |  | -0.45 (-2.66 to 1.76) | 0.520 |  | -0.42 (-3.24 to 2.40) | 0.771 |
| Apgar score (1 min) | 1.59 (1.41 to 1.77) | 0.001 |  | 1.58 (1.40 to 1.76) | 0.001 |  | 1.27 (1.06 to 1.48) | 0.001 |
| Apgar score (5 min) | 0.39 (0.27 to 0.51) | 0.001 |  | 0.38 (0.26 to 0.49) | 0.001 |  | 0.30 (0.15 to 0.46) | 0.001 |
| Apgar score (10 min) | 0.16 (0.05 to 0.26) | 0.003 |  | 0.15 (0.05 to 0.26) | 0.004 |  | 0.14 (-0.01 to 0.27) | 0.040 |
| Asphyxia_neonatal | 0.17 (0.09 to 0.29) | 0.001 |  | 0.16 (0.09 to 0.28) | 0.001 |  | 0.17 (0.09 to 0.35) | 0.001 |
| Admission to NICU | 0.33 (0.24 to 0.44) | 0.001 |  | 0.32 (0.24 to 0.44) | 0.001 |  | 0.42 (0.26 to 0.68) | 0.001 |

*Adjusted for anesthesia-to-delivery time (min)

**Adjusted for anesthesia-to-delivery time (min), and relevant confounding factors (gestational weeks, gravity, anterior placenta, previous cesarean delivery, previous placenta previa, antepartum hemorrhage, and emergency cesarean delivery).
